# Disruption of the Rice Plastid Ribosomal Protein S20 Leads to Chloroplast Developmental Defects and Seedling Lethality

**DOI:** 10.1534/g3.113.007856

**Published:** 2013-10-01

**Authors:** Xiaodi Gong, Quan Jiang, Jianlong Xu, Jianhui Zhang, Sheng Teng, Dongzhi Lin, Yanjun Dong

**Affiliations:** * Development Center of Plant Germplasm Resources, College of Life and Environmental Sciences, Shanghai Normal University, Shanghai 200234; † Institute of Crop Sciences, Chinese Academy of Agricultural Sciences, Beijing, 100081; ‡ Institute of Plant Physiology and Ecology, Shanghai Institute for Biological Sciences, Chinese Academy of Sciences, Shanghai 200032, China

**Keywords:** albino, chloroplast development, lethality, *Oryza sativa*, plastid ribosomal protein (PRP)

## Abstract

Plastid ribosomal proteins (PRPs) are essential for ribosome biogenesis, plastid protein biosynthesis, chloroplast differentiation, and early chloroplast development. This study identifies the first rice PRP mutant, *asl1* (*albino seedling lethality1*), which exhibits an albino lethal phenotype at the seedling stage. This albino phenotype was associated with altered chlorophyll (Chl) content and chloroplast development. Map-based cloning revealed that *ASL1* encodes PRP S20 (PRPS20), which localizes to the chloroplast. *ASL1* showed tissue-specific expression, as it was highly expressed in plumule and young seedlings but expressed at much lower levels in other tissues. In addition, *ASL1* expression was regulated by light. The transcript levels of nuclear genes for Chl biosynthesis and chloroplast development were strongly affected in *asl1* mutants; transcripts of some plastid genes for photosynthesis were undetectable. Our findings indicate that nuclear-encoded PRPS20 plays an important role in chloroplast development in rice.

The chloroplast is a semiautonomous organelle and a central node in the metabolic network in photosynthetic cells of plants. Photosynthesis and other metabolic processes occur in chloroplasts, including the syntheses of key metabolites such as tetrapyrroles, terpenoids, lipids, amino acids, and hormones (Kusumi *et al.* 2011). During the formation of green tissues, mature chloroplasts develop from proplastids and divide to maintain their numbers within the cell. Chloroplast development from proplastids can be separated into three steps, which are coordinately regulated by plastid and nuclear genes (Mullet 1993; Kusumi *et al.* 2010, 2011). In the first step, plastid replication and plastid DNA synthesis become activated. The second step is the chloroplast “build-up” step, characterized by the establishment of the chloroplast genetic system. In this step nuclear-encoded plastid RNA polymerase, termed NEP, preferentially transcribes plastid genes encoding the plastid gene expression machinery (Hajdukiewicz *et al.* 1997), and transcription and translation in the chloroplast dramatically increases. In the final step, the plastid and nuclear genes encoding the photosynthetic apparatus are expressed at very high levels. Plastid genes for the photosynthetic apparatus mainly are transcribed by the plastid-encoded RNA polymerase, termed PEP (MacIossek *et al.* 1999). The expression of these genes results in the synthesis and assembly of the photosynthetic apparatus.

Plastid ribosomal proteins (PRPs) are crucial for the establishment of the transcription/translation apparatus during the build-up step of chloroplast differentiation (Schultes *et al.* 2000). The lack of PRPs has diverse phenotypic effects in plants, including lethality, decreased photosynthetic capacity, and reduced plant height (Romani *et al.* 2012). Bacteria and chloroplasts translate via 70S ribosomes and most PRPs have orthologs in bacteria. For example, the chloroplast small (30S) ribosomal subunit consists of 21 subunits and the large (50S) subunit consists of 31 subunits, which all have orthologs in *Escherichia coli*. In the chloroplast 30S subunit, plastid genes encode 12 subunits, and nuclear genes encode 9 subunits; similarly, in the 50S subunit, plastid and nuclear genes encode 9 and 22 subunits, respectively (Yamaguchi and Subramanian 2000). Although much is known about the composition of the chloroplast ribosome, the molecular basis of the mechanisms that initiate and control this process in higher plants remain basically unknown.

Schultes *et al.* (2000) identified the first higher plant PRP mutant (*hcf60*) in maize, where *hcf60* mutants show an unstable pale green seedling lethal phenotype, caused by a defective nuclear-encoded chloroplast ribosomal small subunit protein 17 (RPS17). More recently, in *Arabidopsis*, four of the small subunits (RPS5, 9, 13, and 20) and 10 of the large subunits (RPL1, 4, 6, 10, 13, 18, 21, 27, 31, and 35) were reported to be essential for embryogenesis (Hsu *et al.* 2010; Bryant *et al.* 2011; Muralla *et al.* 2011; Lloyd and Meinke 2012; Romani *et al.* 2012; Yin *et al.* 2012b). Also, the large subunit protein RPL28 is essential at the latest stage of embryo-seedling development during the greening process (Romani *et al.* 2012). Although abolishing plastid protein biosynthesis is lethal, each individual component of the plastid ribosome may not be essential. For example, RPS1, 17, and 24 appear not to be required for basal ribosome activity; plastid protein synthesis and photosynthesis are perturbed in *rps1*, *rps17*, and *rpl24* plants, but the organism can complete its entire life cycle in their absence (Romani *et al.* 2012). Despite this, only few PRP mutants have had their gene−phenotype relationships unambiguously confirmed by allelism tests or genetic complementation assays.

Here we report the first PRP mutant from rice, *asl1*, which has an albino seedling lethal phenotype and is defective in the nuclear gene encoding plastid ribosomal protein S20 (PRPS20). Our findings show that PRPS20 plays an essential role in the development of chloroplasts and the establishment of the transcription/translation apparatus in rice.

## Materials and Methods

### Plant materials and growth conditions

The rice albino seedling lethal mutant *asl1* was derived from a ^60^Co gamma ray irradiated mutant pool of *Oryza sativa* cultivar Jiahua1 (wild type; WT). To generate a large F_2_ population for genetic analysis, heterozygous plants (*ASL1*/*asl1*) were crossed with the indica cultivar Pei’ai64S. Surface-sterilized germinated seeds of WT and *asl1* plants were sown in natural rice-paddy soil in plastic pots. Plants were grown in a growth chamber under 12 hr of light and 12 hr of dark at a constant temperature of 32° and humidity of approximately 70%. Mutant segregants were distinguished from the normal segregants by their albino phenotype.

### Cloning of *ASL1*

To map the *ASL1* gene, 3478 mutants were selected from an F_2_ population derived from a cross between heterozygous plants (*ASL1*/*asl1)* and Pei’ai64S. Genomic DNA was extracted from F_2_ plants with use of the CTAB method and analyzed for cosegregation using available simple sequence repeat markers (McCouch *et al.* 2002). New insertion-deletion markers were developed based on the entire genomic sequences of a Nipponbare variety (Goff *et al.* 2002) and an indica variety 93−11 (Yu *et al.* 2002). The primers for genotyping markers were designed using PRIMER 5.0, and the markers are listed in Supporting Information, Table S1. Gene prediction was performed using the Rice Genome Annotation Project (http://rice.plantbiology.msu.edu/cgi-bin/gbrowse/rice/). The genomic DNA fragments of candidate genes were amplified from mutant and WT plants and sequenced. The sequencing reactions were performed by Sinogenomax Co., Ltd.

### Complementation of the *asl1* mutant

A 4.03-kb genomic DNA fragment was amplified from WT plants with the primers 5′-AAAAGGATCCCGGAAGCGGAAGTAGAAG-3′ and 5′-AAAAGTCGACCATTGATGACCACGGTGT-3′. This fragment contains the entire *ASL1* coding region, the 1352-bp upstream sequence and the 545-bp downstream sequence, and was inserted into the binary vector pCAMBIA1301. The resultant plasmid (pCAMBIA1301-ASL1) and the empty vector pCAMBIA1301 were introduced into the *asl1* mutant by *Agrobacterium tumefaciens*−mediated transformation (Hiei *et al.* 1994). Transgenic plants were selected on hygromycin medium, and were further verified by PCR amplification using specific primers (5′-AAAAGTCGACTGGGTCCTATCCTCCTA-3′ and 5′-AATTGTCGACTTCTTAGATGGGCTGGT-3′).

### Measurement of chlorophyll (Chl) and carotenoids (Car)

Chl and Car contents were determined following the method of Arnon (1949). Leaves (approximately 0.02 g fresh weight) were cut and soaked in 5 mL of acetone (5): ethanol (4): H_2_O (1) miscible liquids for 18 hr in the dark, then residual plant debris was removed by centrifugation. The supernatants were analyzed with a DU 800 UV/Vis Spectrophotometer (Beckman Coulter) at 665, 649 and 470 nm, respectively.

### Transmission electron microscopy (TEM)

For TEM analysis, top leaves were sampled from three-leaf-stage seedlings grown in a paddy field and transverse leaf sections were fixed in 2.5% glutaraldehyde and then in 1% OsO_4_. After staining with uranyl acetate, tissues were further dehydrated in an ethanol series and finally embedded in Spurr’s medium before ultrathin sectioning. Samples were stained again and examined with a Hitachi-7650 transmission electron microscope.

### Phylogenetic analysis

The BLAST search program (www.ncbi.nlm.nih.gov/BLAST/) was used to search for the sequences of proteins homologous to ASL1. The sequences of RPS20 domains were aligned using BioXM version 2.6 and the neighbor-joining tree was generated by the Poisson correction method with MEGA version 5.1. Bootstrap replication (1000 replications) was used for statistical support for the nodes in the phylogenetic tree.

### Subcellular localization

To investigate the subcellular localization of ASL1, the *ASL1* ORF was amplified from WT plants using primers 5′-GGAAGATCTATGGCGACCGCCACCTCC-3′ and 5′-CGGGGTACCCCGCTGGTAGCAGCAGGTTC-3′ and introduced into vector pMON530-GFP at the *Kpn*I and *Bgl*II sites (Yin *et al.* 2012a). Transgenic tobacco leaves were observed under a laser scanning microscope (Leica TCS SP2). The GFP fluorescence images were obtained using an argon ion laser with excitation at 488 nm and a 505- to 530-nm band-pass filter. Chl autofluorescence was detected with a 570-nm filter.

### Reverse transcription polymerase chain reaction (RT-PCR) and quantitative real-time PCR analysis

Total RNA was extracted from seedling roots, young culms, plumules, young leaves, flag leaves, and young panicles using an RNA Prep Pure Plant kit (Tiangen Co., Beijing, China) and was reverse transcribed using a SuperScript II kit (TaKaRa). Real-time PCR was performed using a SYBR Premix Ex TaqTM kit (TaKaRa) on an ABI prism 7900 Real-Time PCR System. The 2^-ΔΔC^T method was used to analyze the relative changes in gene expression (Livak and Schmittgen 2001). The primers for *ASL1*, Chl biosynthesis, photosynthesis, and chloroplast development −associated genes (*HEMA1*, *CAO1*, *Cab1R*, *YGL1*, *PORA*, *rbcS*, *rbcL*, *psaA*, *psbA*, *RNRS*, *RNRL*, *V2*, *OsRpoTp*, *OsPoLP1*, *FtsZ*, *16S rRNA*, *RpoB*, and *RPS7*) are listed in Table S2. The rice *Actin* gene was used as a reference.

## Results

### Characterization of the *asl1* mutant

**Figure 1:**
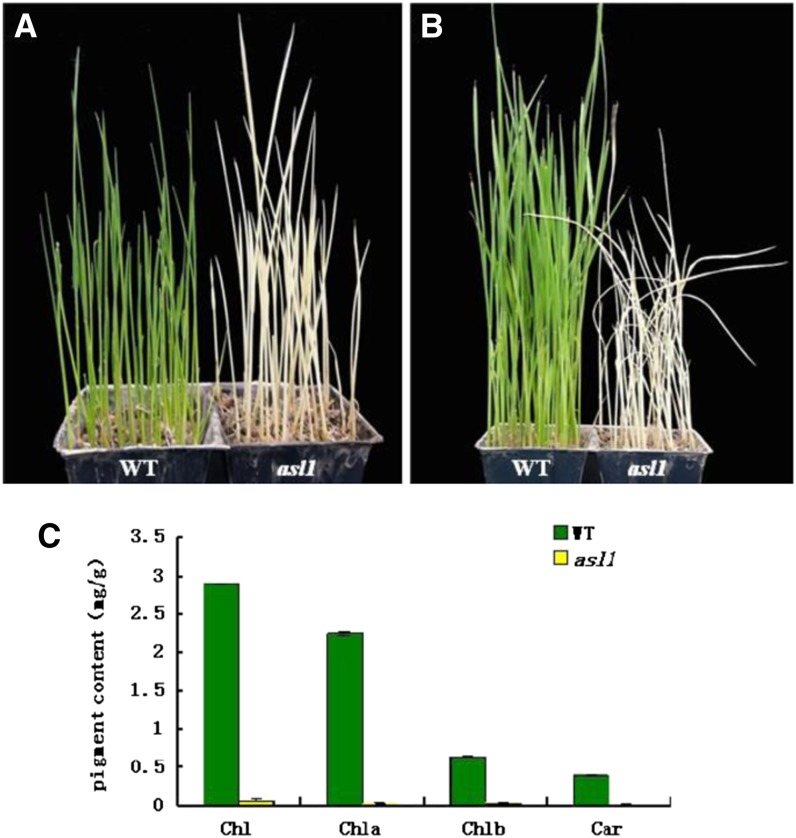
Characterization of the *asl1* mutants: (A) One-week-old plants; (B) Three-week-old plants; (C) The pigment contents in leaves of 1-wk-old *asl1* mutants are much lower than that in Jiahua1 (wild type; WT) Chlorophyll a (Chla), chlorophyll b (Chlb), total chlorophyll (Chl), and carotenoid (Car).

The *asl1* mutant is an albino, seedling-lethal mutant isolated from *japonica* rice Jiahua1 irradiated with ^60^Co gamma rays. Although *asl1* seeds germinated, all leaves of mutant plants exhibited an albino phenotype (Figure 1A) and the seedlings did not survive past the four-leaf stage (Figure 1B). In addition, the contents of Chl a, Chl b, and Car approached zero in *asl1* mutant plants (Figure 1C). These results suggested that the albino phenotypes of the young *asl1* seedlings are caused by the reduction in total Chl content.

**Figure 2:**
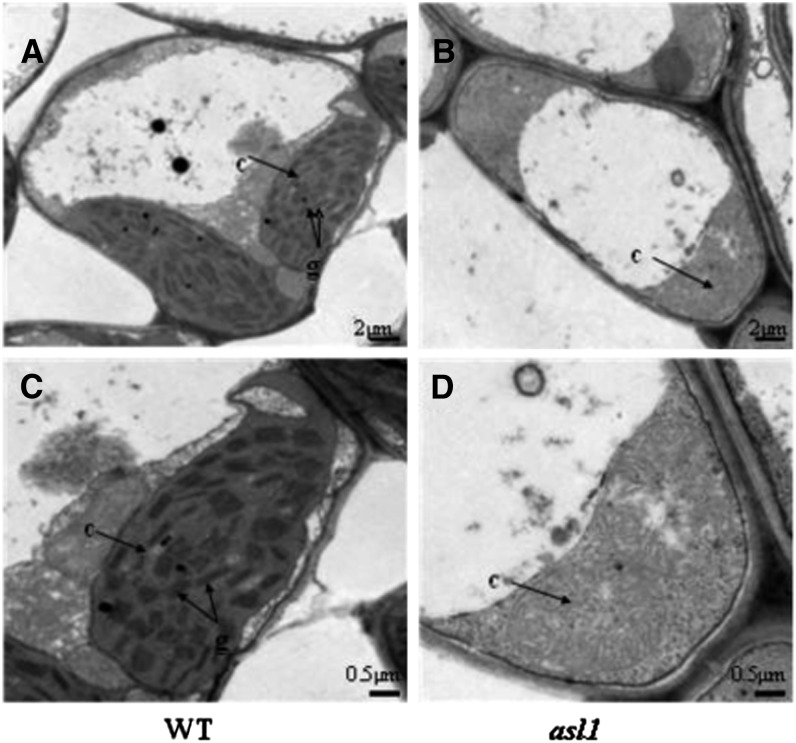
TEM of chloroplasts in expanded third leaves. (A) The cell of WT; (B) the cell of an *asl1* mutant; (C) an intact chloroplast in the WT cell; and (D) an abnormal chloroplast in the *asl1* mutant cell. c, chloroplast; g, grana stack.

To examine the status of chloroplasts in the *asl1* mutants, we examined the ultrastructure of chloroplasts at the three-leaf stage by TEM. Normal chloroplasts were found in all WT plants, with grana stacks that were dense and well structured (Figure 2, A and C). By contrast, in *asl1* mutants, normal chloroplasts were not observed and chloroplast development appeared to cease at the proplastid stage (Figure 2, B and D). These observations indicate that the *asl1* mutation affects chloroplast development.

### Map-based cloning of the *ASL1* gene

To understand the molecular mechanism responsible for the phenotype of *asl1* mutant, we used map-based cloning to identify the *ASL1* locus. No seeds could be obtained from homozygous *asl1* plants because of the seedling-lethal phenotype. Therefore, to generate a population for mapping, we crossed heterozygous *ASL1/asl1* plants with indica cultivar Pei’ai64S. The F_1_ plants (*ASL1/ASL1*: *ASL1/asl1* = 1:1) from the crosses were all normal green; however, the F_2_ plants selfed from the heterozygous F_1_ plants (*ASL1/asl1*) segregated 3:1 (green: albino = 298:83; χ2 = 1.93; *P* > 0.05), indicating the albino phenotype is a recessive trait controlled by a single gene, *asl1*.

**Figure 3:**
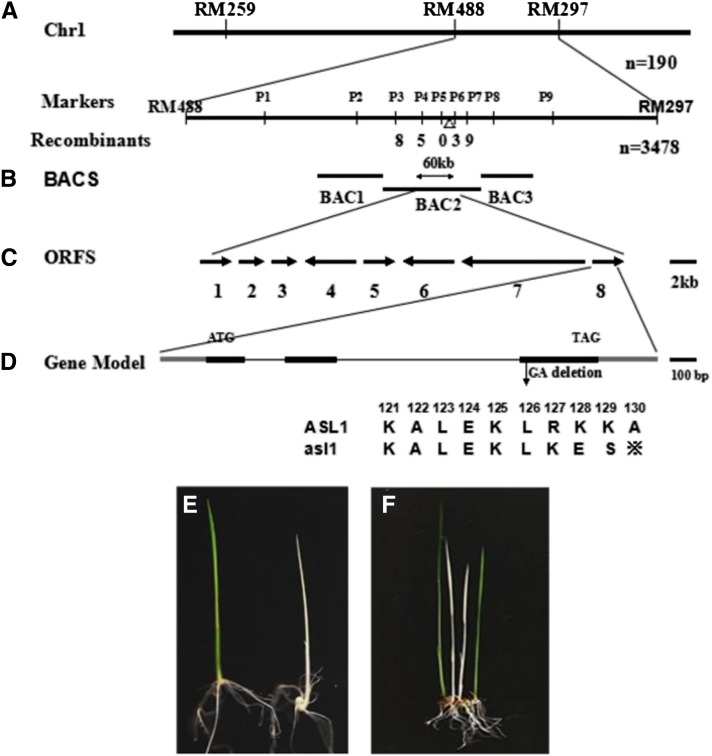
Map-based cloning of *ASL1*: (A) The *ASL1* locus initially was mapped to a region between markers RM488 and RM297 on the long arm of rice chromosome 1 (Chr1) with 190 recessive individuals. (B) Fine mapping of *ASL1* to BAC2 (AP003335) within a 60-kb region by the markers P4 and P6 using 3478 recessive individuals. (C) Diagram of the predicted ORFs and the mutation site. A 2-bp deletion (GA) in ORF8 results in a premature stop codon; (D) Gene model of *ASL1*. (E) Complementation of the *asl1* mutant. The *asl1* mutants transformed with pCAMBIA1301-ASL1 vector (left) show normal green leaves, whereas the mutants transformed with empty pCAMBIA1301 vector (right) have albino leaves. (F) T_1_ of transgenic plants.

For mapping the *ASL1* gene, we selected 3478 albino mutant plants from F_2_ populations. Using 190 mutant individuals, the *ASL1* locus was initially mapped to the long arm of chromosome 1 between the markers RM488 and RM297. Then nine insertion-deletion markers were developed between RM488 and RM297, and no recombinant near the marker P5 was detected (Figure 3A). The *ASL1* locus was further narrowed down to a 60-kb region on BAC2 (AP00333; Figure 3B), including eight putative open reading frames (ORFs; http://rice.plantbiology.msu.edu; Figure 3C). We sequenced all putative ORFs and found a 2-bp deletion in *LOC-Os01g48690*; this deletion causes a premature stop codon (Figure 3D). The 2-bp deletion in the *asl1* transcript was not present in the WT parents (Jiahua 1).

To confirm that the loss of *ASL1* is responsible for the *asl1* mutant phenotype, we complemented the mutant phenotype with a wild-type genomic fragment. A 4.03-kb genomic DNA fragment containing the entire *ASL1* gene, 1352 bp of upstream sequence, and 545 bp of downstream sequence, was cloned into the vector pCAMBIA1301. The resultant plasmid, pCAMBIA1301-ASL1, was then transformed into *asl1* mutant callus via *Agrobacterium tumefaciens*-mediated transformation (Hiei *et al.* 1994). Eleven independent transgenic lines transformed with pCAMBIA1301-ASL1 were identified (Figure 3E), and they showed complementation of the *asl1* phenotype. Segregation occurred in the seeds of transgenic plants (T_1_) and all green seedlings contained the transgene (Figure 3F). As controls, 18 independent lines transformed with empty vector, pCAMBIA1301, all failed to complement the *asl1* mutant phenotype (Figure 3E). These results confirmed that *LOC-Os01g48690* is the *ASL1* gene.

### Characterization of the predicted ASL1 protein

Searching the rice genome database revealed that *ASL1* is a single-copy gene with an ORF of 591 nucleotides. The coding region of *ASL1* has three exons (Figure 3D) and encodes a predicted 196-amino acid protein with a calculated molecular mass of 21.52 kD. Database searches using Pfam (Finn *et al.* 2006) revealed that *ASL1* encodes the chloroplast 30S ribosomal protein S20 (PRPS20). The N-terminal portion of ASL1/PRPS20 protein contains a DUF3782 domain, and the C-terminus contains a ribosomal protein S20 domain. The *asl1* mutation occurs in the ribosomal protein S20 domain.

**Figure 4:**
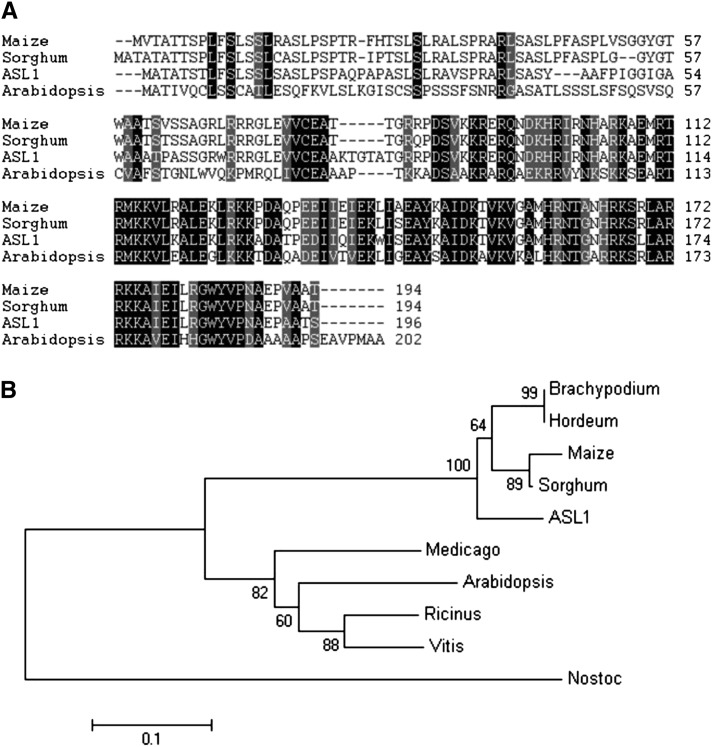
Phylogenic analysis of ASL1 protein. (A) Amino acid sequence alignment of the four kinds of RPS20 proteins. Amino acids fully or semiconserved are shaded black and gray, respectively. (B) Homologous proteins similar to ASL1 were used to obtain a phylogenetic tree with the program Mega5.1, which was bootstrapped over 1000 cycles. Significance values greater than a 50% cutoff threshold are indicated near the relative branches.

We identified orthologs of ASL1 from *Arabidopsis thaliana*, *Zea mays*, *Medicago truncatula*, *Sorghum bicolor*, *Hordeum vulgare*, *Brachypodium dystachion*, and *Nostoc Vauch* in the National Center for Biotechnology Informationj database. Among these, ASL1 protein exhibited the greatest sequence similarity to RPS20 in *Brachypodium*, with 84% amino acid identity, and it shared 69% amino acid identity with RPS20 from *Arabidopsis* (Figure 4A). These data indicated that the ASL1 protein is very conserved in greater plants. To investigate the evolutionary relationship between ASL1 homologs, a phylogenic analysis was performed. As shown in Figure 4B, the proteins from monocots and dicots clustered in different subclades. Our data indicated that ASL1/PRPS20 is conserved in photosynthetic organisms. However, its function in higher plants remains unclear.

### *ASL1* expression

**Figure 5:**
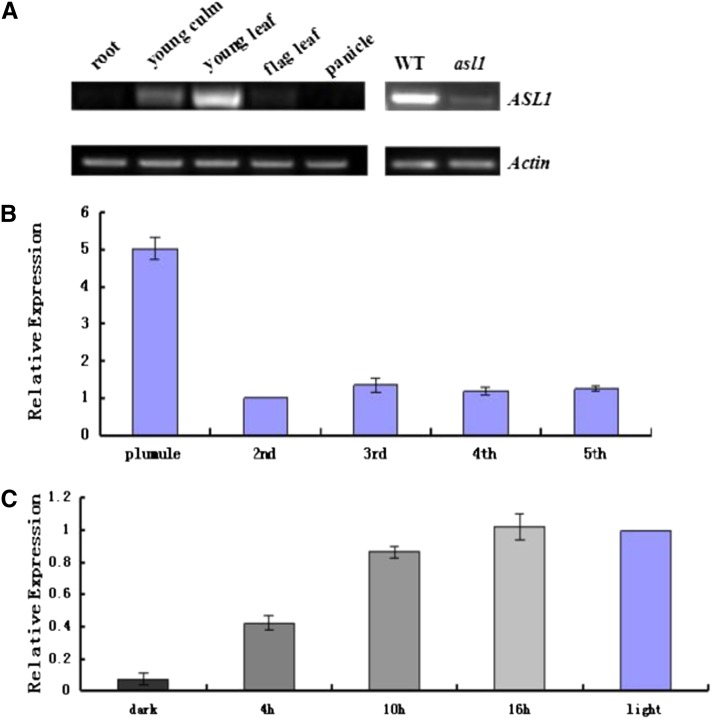
Expression analysis of *ASL1:* (A) RT-PCR analysis of *ASL1* in root, young culm, young leaf, flag leaf and panicle of WT, the leaf of WT, and *asl11*. Rice *Actin* gene was used as a control. (B) Transcript levels of *ASL1* in top leaves sampled from the two-, three-, four- and five-leaf stages and plumule. The *ASL1* transcript level in top leaves of the two-leaf stage was set to 1.0, and the relative values in other treatments were calculated accordingly. (C) Expression of *ASL1* under illumination from 4 to 16h. The *ASL1* transcript level in seedlings growing under illumination was set to 1.0, seedlings grown under continuous light or dark were chosen as the control.

We used RT-PCR to examine the expression of *ASL1* in WT plants (Figure 5A). We detected a significantly high level of expression in young leaves, culms, and flag leaves but weak expression in roots and young panicles. The *ASL1* transcript was substantially reduced in *asl1* mutants, indicating that the mutation in *ASL1* causes a decreased transcript level.

To further investigate the expression of *ASL1* during seedling-growth stages, we performed quantitative real-time RT-PCR. *ASL1* expression peaked in the plumule and declined to a relatively low level in the second to fifth leaf (Figure 5B). These observations suggested that *ASL1* plays an important role in rice seedlings, especially at early stages.

Light induces the differentiation of non-photosynthetic proplastids into fully functional, photosynthetic chloroplasts (Su *et al.* 2012). To examine the effects of light on the expression of *ASL1*, we used real-time PCR to measure *ASL1* expression in 10-d-old WT (Jiahua1) etiolated seedlings exposed to light for 0, 4, 10, and 16 hr. RNA from the seedlings grown in standard light intensity for 10 d was used as the control. *ASL1* transcript levels were high under light and much lower in the dark in 10-d-old etiolated seedlings (Figure 5C). Furthermore, *ASL1* expression was highly induced after illumination for 4 hr, and it reached maximal levels after 16 hr of illumination. These observations suggest that light plays a major role in regulating *ASL1* expression.

### The *asl1* mutant affects expression of related genes

**Figure 6:**
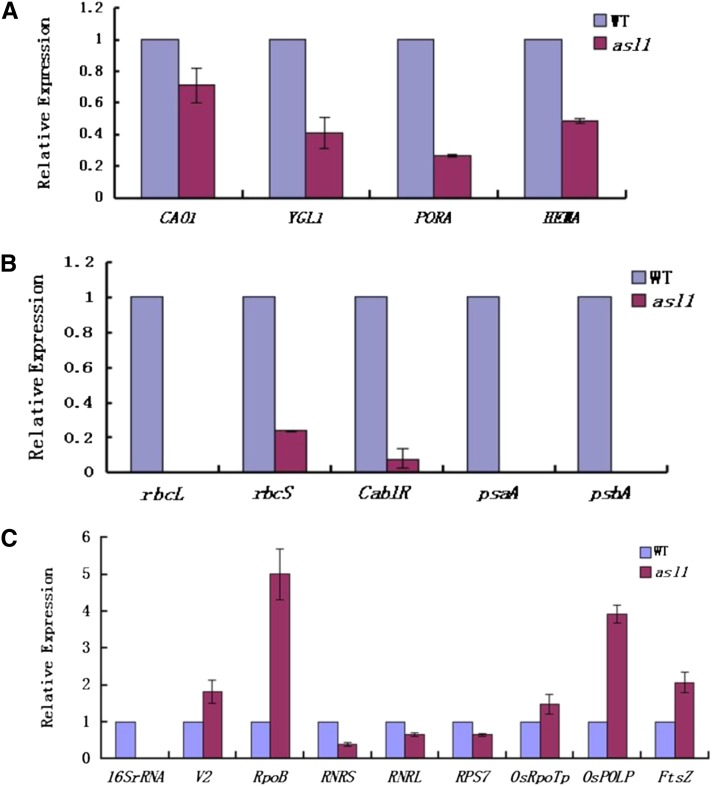
Expression analysis of genes associated with Chl biosynthesis, photosynthesis, or chloroplast development by real-time PCR. (A) Expression of genes associated with Chl biosynthesis. (B) Expression of genes associated with photosynthesis. (C) Expression of genes associated with chloroplast development. The relative expression level of each gene was normalized using *Actin* as an internal control. The expression level of each gene at the three-leaf stage in Jiahua1 was set as 1.0 and other samples were calculated accordingly. Error bars (SDs) are based on three independent experiments.

We next examined the transcript levels of genes associated with Chl biosynthesis, photosynthesis, and chloroplast development in *asl1* mutant and WT plants. The expression of genes required for Chl biosynthesis (Wu *et al.* 2007), such as *HEMA1* (encoding glutamyl tRNA reductase), *CAO1* (CHLOROPHYLLIDE A OXYGENASE1), *YGL1* (encoding a Chl synthetase), and *PORA* (encoding NADPH-dependent protochlorophyllide oxidoreductase) were obviously down-regulated in *asl1* mutants (Figure 6A), resulting in a reduction in Chl content (Figure 1C). The photosynthesis-associated transcripts of plastid genes, *psaA* and *psbA* (encoding two reaction center polypeptides) and *rbcL* (encoding the large subunit of Rubisco), were undetectable. Expression of the nuclear genes, *Cab1R* (encoding the light harvesting Chla/b-binding protein of PSII) and *rbcS* (encoding the small subunit of Rubisco (Kyozuka *et al.* 1993), was also significantly reduced in *asl1* mutants (Figure 6B). Decreased or absent expression of so many key genes can impair photosynthesis and finally lead to the death of the mutant seedlings.

For chloroplast development-associated transcripts, we examined the expression of the nuclear-encoded genes *RNRS* and *RNRL*, encoding the large and small subunits of ribonucleotide reductase (Yoo *et al.* 2009), V_2_, encoding plastidal guanylate kinase (Sugimoto *et al.* 2007), *OsRpoTp*, encoding NEP core subunits (Hiratsuka *et al.* 1989), *OsPoLP1*, encoding one plastidal DNA polymerase (Vitha *et al.* 2001), and *FtsZ*, encoding a component of the plastid division machinery (Takeuchi *et al.* 2007). We also examined the plastid-encoded genes 16S rRNA (16S ribosomal RNA), *RpoB* encoding one PEP core subunit (Inada *et al.* 1996; Kusumi *et al.* 2011) and *RPS7* encoding one ribosomal protein (Kusumi *et al.* 2011). We found that in the *asl1* mutant plants *V_2_*, *OsRpoTp*, *OsPoLP1*, *FtsZ*, and *RpoB* were up-regulated, whereas *RNRS*, *RNRL*, *16S rRNA*, and *RPS7* were severely suppressed (Figure 6C). Overall, these observations indicated that the *asl1* mutation affects the transcription of genes associated with not only Chl biosynthesis (Figure 6A) and photosynthesis (Figure 6B) but also with early chloroplast development.

### Subcellular localization of ASL1 protein

**Figure 7:**
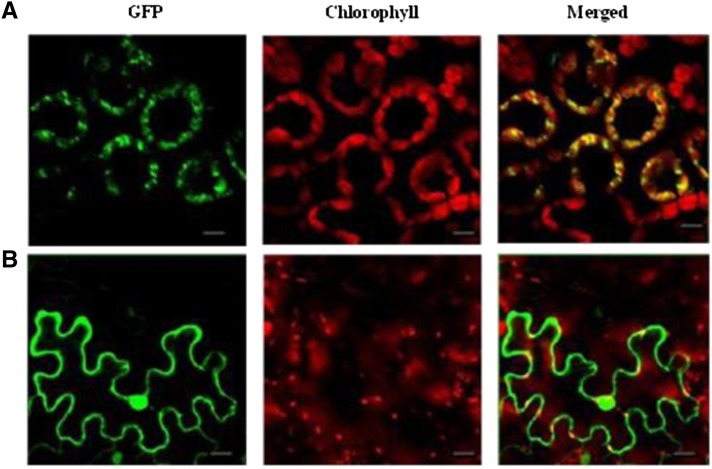
Subcellular localization of the ASL1 protein. (A) A tobacco mesophyll cell expressing ASL1–GFP; (B) A tobacco epidermal cell expressing GFP alone. The scale bar represents 20 μm.

The ASL1 protein was predicted to localize to chloroplasts by ChloroP (Emanuelsson *et al.* 1999) and TargetP (Emanuelsson *et al.* 2000). To determine the actual subcellular localization of ASL1, we constructed an ASL1-GFP fusion protein. To that end, a full-length *ASL1* coding sequence lacking a stop codon was fused to the GFP coding sequence, the fusion gene was transformed into tobacco cells, and confocal microscopy was used to observe the fluorescent signal 48 hr after transformation. GFP alone was used as a control. The green fluorescent signal of ASL1-GFP colocalized with the Chl autofluorescence in the chloroplasts (Figure 7A), suggesting that ASL1 protein is localized to the chloroplast. By contrast, the epidermal cells transformed with the empty GFP vector without a specific targeting sequence had green fluorescent signals in the plasma membrane and the nucleus (Figure 7B).

## Discussion

In this study, we cloned the rice nuclear-encoded PRP gene, *ASL1*, by a map-based cloning strategy using the single recessive mutant *asl1*, which exhibits an albino seedling lethal phenotype (Figure 1, A and B). TEM analysis indicated that chloroplast development is seriously impaired in the *asl1* mutant (Figure 2, B and D). The *asl1* mutant had a 2-bp deletion in the third exon of *ASL1* (*LOC-Os01g0678600*), resulting in a premature stop codon (Figure 3D). Molecular complementation verified that the *asl1* mutant phenotype is attributable to the 2-bp deletion in *ASL1* encoding PRPS20, a protein belonging to the PRP family.

### The light-regulated PRP gene *ASL1* is essential for chloroplast development in rice

The PRPs originated from the cyanobacterial genome and most protein constituents, including PRPS20, have orthologs in bacteria (Yamaguchi and Subramanian 2000). The high amino acid sequence similarity of ASL1 with the *E. coli* and *Nostoc* proteins (Figure S1) suggested that RPS20 was highly conserved during evolution. In this study, the lack of rice *ASL1* encoding rice PRPS20 leads to an albino seedling phenotype and lethality after the four-leaf seedling stage (Figure 1B), a phenotype that can be attributed to impaired chloroplast development (Figure 2, B and D), Chl biosynthesis (Figure 1C), and photosynthesis. These observations suggest that *ASL1* is essential for chloroplast development in rice.

Previous studies reported the loss of *E. coli* RPS20 function not only influenced the properties of the ribosome, but also changed the modification pattern of *16S rRNA*, which led to an inability of the mutant to associate its 30S subunits with 50S subunits to form 70S ribosomes (Aulin *et al.* 1993). *Arabidopsis* PRPS20 was also reported to be required for basal ribosome activity, consistent with the results in this study, based on the severe hindrance of 16S rRNA expression (Figure 6C); however, in *Arabidopsis* the lack of PRPS20 leads to embryo lethality (Romani *et al.* 2012) rather than the albino seedling lethality we observed in rice. The phenotypic difference may be due to different pathways regulating embryogenesis in *Arabidopsis* and rice or to a different mutation site. Similar results were reported in previous studies. For instance, one *maize* PRP protein mutant, *prps17*, shows an unstable pale green seeding lethal phenotype (Schultes *et al.* 2000), but *Arabidopsis* mutant *prps17* can complete its entire life cycle, indicating that *Arabidopsis* PRPS17 appears to be dispensable for basal ribosome activity (Romani *et al.* 2012). However, Romani *et al.* (2012) also reported that the *Arabidopsis PRPL28* was essential for the greening of embryos and seedlings. Interestingly, the *prpl28* mutant had a similar phenotype to the *asl1* mutant. In view of the higher expression of *ASL1* in plumule than in seedlings and other tissues (Figure 5, A and B) and chloroplast-localization of ASL1 (Figure 7A), ASL1 protein may also play a role in the greening process in early rice development.

Previous studies showed that light affects the expression of some genes in chloroplast development (Tobin and Silverthorne 1985; Zhao *et al.* 1999; Su *et al.* 2012). The increased accumulation of *ASL1* transcript after light illumination (Figure 5 C) in this study also affirmed the major role of light during chloroplast development.

### ASL1 is involved in the establishment of the transcription/translation apparatus and plastid-to-nucleus signaling

Chloroplast differentiation and development can be divided into three steps in higher plants: proplastid growth and activation of plastid DNA synthesis, chloroplast “build-up,” and high-level expression of plastid and nuclear genes encoding the photosynthetic apparatus (Chory 1991; Mullet 1993). The transcriptional machinery plays an important role in chloroplast differentiation. Transcription of plastid genes is regulated by at least two RNA polymerases (PEP and NEP) (Hess and Börner 1999). PEP is a multisubunit eubacteria-type RNA polymerase and NEP is a single-subunit bacteriophage-type enzyme (Liere and Maliga 2004). In previous studies, transcription patterns in PEP-deficient plants indicated that NEP mainly transcribes plastid genes for the transcription/translation apparatus, whereas PEP functions in the expression of photosynthesis-related genes (Kapoor *et al.* 1997; Liere and Maliga 1999). Once PEP becomes active, NEP is assumed to be a relatively minor player required to maintain the levels of ribosomes, tRNAs and PEP (Liere and Maliga 2004).

In this study, we found that the *asl1* mutation disrupts the expression of plastid and nuclear genes associated with chloroplast development. The transcripts for PEP components (*RpoB*) accumulated to a high level (Figure 6C). However, transcripts of PEP-transcribed plastid genes (*psaA*, *psbA*) were severely reduced (Figure 6B), suggesting that in *asl1* mutants, accumulation of transcripts for PEP components did not result in the formation of functional PEP due to the disruption of the transcription/translation apparatus. The transcripts of *rbcL*, an indicator for the accumulation of polysomes (Barkan 1993), and chloroplast 16S rRNA, were undetectable and *RPS7* transcripts were severely suppressed in *asl1* mutants (Figure 6, B and C). This finding indicates that the *asl1* mutation affects the assembly and accumulation of plastid ribosomes, resulting in disruption of plastid translation during chloroplast development. Thus, ASL1 plays an important role, not only in plastid translation, but also in the assembly and the accumulation of plastid ribosomes. In addition, in *asl1* plants, some nuclear genes mainly expressed at the early stage of chloroplast differentiation before PEP becomes active (Inada *et al.* 1996) such as *V_2_*, *OsRpoTp*, *OsPoLP1*, and *FtsZ* were distinctly up-regulated (Figure 6C). This may be a feedback mechanism if cells try to increase PEP levels to transcribe photosynthetic genes by up-regulating the transcriptions of these genes. Similar results were demonstrated in *v3* and *st1* rice mutants (Yoo *et al.* 2009). In addition, the decrease in transcripts of genes for Chl biosynthesis (*CAO1*, *YGL1*, *PORA*, and *HEMA*; Figure 6A) is probably due to the hindrance of chloroplast development.

Furthermore, the expression of plastid translation machinery is essential for subsequent expression of nuclear-encoded chloroplast proteins. Some of these signaling pathways are mediated by tetrapyrrole accumulation or a change of chloroplast redox state, which are required for the proper expression of several nuclear genes, including *Cab1R* and *rbcS* (Surpin *et al.* 2002; Strand *et al.* 2003). Because a decrease in *Cab1R* and *rbcS* transcripts also was observed in this study (Figure 6B), it can be deduced that a change in plastid-to-nucleus signaling in *asl1* mutant affects the expressions of nuclear-encoded genes required for photosynthesis. These results agree with previous studies of a barley ribosome deficient mutant (Hess *et al.* 1993) and the *v2* mutant (Sugimoto *et al.* 2004). Thus, the deficiency of plastid translation at an early stage of chloroplast development leads to unusual expression of nuclear genes for chloroplast proteins at a later stage. This study demonstrates the importance of the nuclear-encoded PRPS20 *ASL1* for plastid-to-nucleus signaling responsive to plastid translation for chloroplast development in rice.

## Supplementary Material

Supporting Information
