# Supplementary material for: Disruption of the Rice Plastid Ribosomal Protein S20 Leads to Chloroplast Developmental Defects and Seedling Lethality
Source: G3 (Bethesda). 2013 Oct 1;3(10):1769–77. doi: 10.1534/g3.113.007856 (PMC3789801; doi:10.1534/g3.113.007856)
Supplement: Supporting Information [file supp_3_10_1769__index.html]

Disruption of the Rice Plastid Ribosomal Protein S20 Leads to Chloroplast Developmental Defects and Seedling Lethality — Supporting Information 

# Disruption of the Rice Plastid Ribosomal Protein S20 Leads to Chloroplast Developmental Defects and Seedling Lethality

## Supporting Information for Gong *et al.*, 2013

**Files in this Data Supplement:**

- Supporting Information - Figure S1 and Tables S1-S2 (PDF, 435 KB)
- Figure S1 - Amino acid sequence alignment of the three kinds of RPS20 proteins (PDF, 364 KB)
- Table S1 - PCR-based molecular markers designed for fine mapping (PDF, 318 KB)
- Table S2 - Primers for Real-time PCR (PDF, 313 KB)
