# Supplementary material for: Disruption of the Rice Plastid Ribosomal Protein S20 Leads to Chloroplast Developmental Defects and Seedling Lethality
Source: G3 (Bethesda). 2013 Oct 1;3(10):1769–77. doi: 10.1534/g3.113.007856 (PMC3789801; doi:10.1534/g3.113.007856)
Supplement: Supporting Information [file supp_g3.113.007856_007856SI.pdf]

G3 · Genes | Genomes | Genetics

Disruption of the Rice Plastid Ribosomal Protein S20 Leads to Chloroplast Developmental Defects and Seedling Lethality

Xiaodi Gong[1], Quan Jiang[1], Jianlong Xu[2], Jianhui Zhang[1], Sheng Teng[3], Dongzhi Lin[1]*, Yanjun Dong[1]*

[1]Development Center of Plant Germplasm Resources, College of Life and Environmental Sciences, Shanghai Normal University, Shanghai 200234, China

[2]Institute of Crop Sciences, Chinese Academy of Agricultural Sciences, 12 South Zhong-Guan Cun Street, Beijing, 100081, China

[3]Institute of Plant Physiology and Ecology, Shanghai Institute for Biological Sciences, Chinese Academy of Sciences, Shanghai 200032, China

*__Corresponding author:__ Development Center of Plant Germplasm Sources, College of Life and Environmental Sciences, Shanghai Normal University, Shanghai 200234, China. E-mail: dzlin@shun.edu.cn and dong@shnu.edu.cn

DOI: 10.1534/g3.113.007856

```
E.coli ------------------------------------------------------------ 0
Nostoc ------------------------------------------------------------ 0
ASL1   MATATSTLFSLSSLSASLPSPAQPAPASLSLRAVSPRARLSASYAAFPIGGIGAWAAATP 60

E.coli ------------------------MANIKSAKKRAIQSEKARKHNASRRSMMRTFIKKVY 36
Nostoc --MDAILVNLVVCVHNYIVFLELTVANTKSALKRAQIAERNRLRNKAYKSAVKTLMKKYF 58
ASL1   ASSGRWRRRGLEVVCEAAKTGTATGRRPDSVKKRERQNDRHRIRNHARKAEMRTRMKKVL 120

E.coli AAIEAG---------DKAAAQKAFNEMQPIVDRQAAKGLIHKNKAARHKANLTAQINKLA 87
Nostoc AAVEVYT--ANPTPESKQAVEERISEAYSKIDKAVKRGVLHPNTGARKKSRLAHKLKPTA 116
ASL1   KALEKLRKKADATPEDIIQIEKWISEAYKAIDKTVKVGAMHRNTGNHRKSLLARRKKAIE 180

E.coli ---------------- 87
Nostoc ---------------- 116
ASL1   ILRGWYVPNAEPAATS 196
```

Figure S1 Amino acid sequence alignment of the three kinds of RPS20 proteins. Amino acids fully or semi-conserved are shaded black and gray, respectively.

Table S1 PCR-based molecular markers designed for fine mapping

Marker	Primer Pairs	Fragment Size (bp)	Originating BAC
P1	AGGGCCTTATATCAAGACACATGC<br>CCACGAACACTCGCATACCC	216	AP003725
P2	GTGTTTGCGAGGGGTCATTA<br>CATTCTCCCATCTATTGCCC	178	AP003706
P3	CAACCTCGTCTTTGAGCCCA<br>CGCCGCAAAATGAGCTCTAC	245	AP003374
P4	TTCATCCTGTCGCCAACG<br>ATGGATGCGTGATGCGTC	152	AP003335
P5	GGAAGGCATAGAACGCAAGA<br>CTGATGCCTATACCATGTCACTGT	122	AP003335
P6	TTCTCCTTCTTCCCGTTCCT<br>CGGCCCTGTACTTCTGATTC	152	AP003335
P7	CAACCAGTCTACAGCCACATCTC<br>AAGGGCGTATTTTCAGCGTC	205	AP003335
P8	CACCCAGAGCAAACTGTCAG<br>GAACTGTACGTTTCCCCCTG	261	AP004363
P9	GCCTTACAACTCAACGACGACT<br>TATGTGTCTAGCTGCACTCCCA	181	AP003450

Table S2 Primers for Real-time PCR

Gene name	Forward primer (5’→3’)	Reverse primer (5’→3’)
Cab1R	AGATGGGTTTAGTGCGACGAG	TTTGGGATCGAGGGAGTATTT
CAO1	GATCCATACCCGATCGACAT	CGAGAGACATCCGGTAGAGC
HEMA	CGCTATTTCTGATGCTATGGGT	TCTTGGGTGATGATTGTTTGG
PORA	TGTACTGGAGCTGGAACAACAA	GAGCACAGCAAAATCCTAGACG
YGL1	CAGTCTCCAATGGCCACCT	TGCTTTCATCAGTGGCTGGT
RPS7	GCCAAAATCCATTCCAATTC	GGAGATGTACACGAGGAGATTG
RpoB	TATGGTCTAATTCCGAGCGGT	TATGGTCTAATTCCGAGCGGT
rbcL	CTTGGCAGCATTCCGAGTAA	ACAACGGGCTCGATGTGATA
psaA	GCGAGCAAATAAAACACCTTTC	GTACCAGCTTAACGTGGGGAG
psbA	CCCTCATTAGCAGATTCGTTTT	ATGATTGTATTCCAGGCAGAGC
OsPoLP	ACCGGTGCTTTCAGGCTTGG	GCTGACTGATAATCACACG
FtsZ	AAAGGACATAACCTTGCAAG	AGTTTTCCTATTGAACCGTG
rbcS	TCCGCTGAGTTTTGGCTATTT	GGACTTGAGCCCTGGAAGG
V2	GAGGAGTTCCTCACGATGAT	AGCATCAATGATAGACTCC
RNRL	GTTAGATGCTTCACTACACAG	GTACCATTGCCAACATGGCAAC
RNRS	GCCAAAATCCATTCCAATTC	GGAGATGTACACGAGGAGATTG
16SrRNA	CCGTTGGTGTTCTTTCCGAT	TTCAAGTCCGCCGTCAAATC
ASL1	CACGCTCTTCTCCCTCTCCT'	GTAGGAGGCGGACAGGCG
OsRpoTp	TCCTCATGTCGAGCAAGGAT	GAAAGAATGTCTGGACTTTG
Actin	AGGAAGGCTGGAAGAGGACC	CGGGAAATTGTGAGGGACAT
