# Supplementary material for: Disruption of the Rice Plastid Ribosomal Protein S20 Leads to Chloroplast Developmental Defects and Seedling Lethality
Source: G3 (Bethesda). 2013 Oct 1;3(10):1769–77. doi: 10.1534/g3.113.007856 (PMC3789801; doi:10.1534/g3.113.007856)
Supplement: Supporting Information [file supp_g3.113.007856_FigureS1.pdf]

```
E.coli ------------------------------------------------------------ 0
Nostoc ------------------------------------------------------------ 0
ASL1   MATATSTLFSLSSLSASLPSPAQPAPASLSLRAVSPRARLSASYAAFPIGGIGAWAAATP 60

E.coli ------------------------MANIKSAKKRAIQSEKARKHNASRRSMMRTFIKKVY 36
Nostoc --MDAILVNLVVCVHNYIVFLELTVANTKSALKRAQIAERNRLRNKAYKSAVKTLMKKYF 58
ASL1   ASSGRWRRRGLEVVCEAAKTGTATGRRPDSVKKRERQNDRHRIRNHARKAEMRTRMKKVL 120

E.coli AAIEAG---------DKAAAQKAFNEMQPIVDRQAAKGLIHKNKAARHKANLTAQINKLA 87
Nostoc AAVEVYT--ANPTPESKQAVEERISEAYSKIDKAVKRGVLHPNTGARKKSRLAHKLKPTA 116
ASL1   KALEKLRKKADATPEDIIQIEKWISEAYKAIDKTVKVGAMHRNTGNHRKSLLARRKKAIE 180

E.coli ---------------- 87
Nostoc ---------------- 116
ASL1   ILRGWYVPNAEPAATS 196
```

Figure S1 Amino acid sequence alignment of the three kinds of RPS20 proteins. Amino acids fully or semi-conserved are shaded black and gray, respectively.
