# Supplementary material for: Disruption of the Rice Plastid Ribosomal Protein S20 Leads to Chloroplast Developmental Defects and Seedling Lethality
Source: G3 (Bethesda). 2013 Oct 1;3(10):1769–77. doi: 10.1534/g3.113.007856 (PMC3789801; doi:10.1534/g3.113.007856)
Supplement: Supporting Information [file supp_g3.113.007856_TableS1.pdf]

Table S1 PCR-based molecular markers designed for fine mapping

Marker	Primer Pairs	Fragment Size (bp)	Originating BAC
P1	AGGGCCTTATATCAAGACACATGC<br>CCACGAACACTCGCATACCC	216	AP003725
P2	GTGTTTGCGAGGGGTCATTA<br>CATTCTCCCATCTATTGCCC	178	AP003706
P3	CAACCTCGTCTTTGAGCCCA<br>CGCCGCAAAATGAGCTCTAC	245	AP003374
P4	TTCATCCTGTCGCCAACG<br>ATGGATGCGTGATGCGTC	152	AP003335
P5	GGAAGGCATAGAACGCAAGA<br>CTGATGCCTATACCATGTCACTGT	122	AP003335
P6	TTCTCCTTCTTCCCGTTCCT<br>CGGCCCTGTACTTCTGATTC	152	AP003335
P7	CAACCAGTCTACAGCCACATCTC<br>AAGGGCGTATTTTCAGCGTC	205	AP003335
P8	CACCCAGAGCAAACTGTCAG<br>GAACTGTACGTTTCCCCCTG	261	AP004363
P9	GCCTTACAACTCAACGACGACT<br>TATGTGTCTAGCTGCACTCCCA	181	AP003450
