# Supplementary material for: Disruption of the Rice Plastid Ribosomal Protein S20 Leads to Chloroplast Developmental Defects and Seedling Lethality
Source: G3 (Bethesda). 2013 Oct 1;3(10):1769–77. doi: 10.1534/g3.113.007856 (PMC3789801; doi:10.1534/g3.113.007856)
Supplement: Supporting Information [file supp_g3.113.007856_TableS2.pdf]

Table S2 Primers for Real-time PCR

Gene name	Forward primer (5’→3’)	Reverse primer (5’→3’)
Cab1R	AGATGGGTTTAGTGCGACGAG	TTTGGGATCGAGGGAGTATTT
CAO1	GATCCATACCCGATCGACAT	CGAGAGACATCCGGTAGAGC
HEMA	CGCTATTTCTGATGCTATGGGT	TCTTGGGTGATGATTGTTTGG
PORA	TGTACTGGAGCTGGAACAACAA	GAGCACAGCAAAATCCTAGACG
YGL1	CAGTCTCCAATGGCCACCT	TGCTTTCATCAGTGGCTGGT
RPS7	GCCAAAATCCATTCCAATTC	GGAGATGTACACGAGGAGATTG
RpoB	TATGGTCTAATTCCGAGCGGT	TATGGTCTAATTCCGAGCGGT
rbcL	CTTGGCAGCATTCCGAGTAA	ACAACGGGCTCGATGTGATA
psaA	GCGAGCAAATAAAACACCTTTC	GTACCAGCTTAACGTGGGGAG
psbA	CCCTCATTAGCAGATTCGTTTT	ATGATTGTATTCCAGGCAGAGC
OsPoLP	ACCGGTGCTTTCAGGCTTGG	GCTGACTGATAATCACACG
FtsZ	AAAGGACATAACCTTGCAAG	AGTTTTCCTATTGAACCGTG
rbcS	TCCGCTGAGTTTTGGCTATTT	GGACTTGAGCCCTGGAAGG
V2	GAGGAGTTCCTCACGATGAT	AGCATCAATGATAGACTCC
RNRL	GTTAGATGCTTCACTACACAG	GTACCATTGCCAACATGGCAAC
RNRS	GCCAAAATCCATTCCAATTC	GGAGATGTACACGAGGAGATTG
16SrRNA	CCGTTGGTGTTCTTTCCGAT	TTCAAGTCCGCCGTCAAATC
ASL1	CACGCTCTTCTCCCTCTCCT'	GTAGGAGGCGGACAGGCG
OsRpoTp	TCCTCATGTCGAGCAAGGAT	GAAAGAATGTCTGGACTTTG
Actin	AGGAAGGCTGGAAGAGGACC	CGGGAAATTGTGAGGGACAT
